# Supplementary material for: Beyond traditional magnetic resonance processing with artificial intelligence
Source: Commun Chem. 2024 Oct 27;7:244. doi: 10.1038/s42004-024-01325-w (PMC11514297; doi:10.1038/s42004-024-01325-w)
Supplement: Supplementary file 1 — Supplementary Material [file 42004_2024_1325_MOESM1_ESM.pdf]

Supplementary of Beyond traditional Magnetic Resonance processing with Artificial intelligence

Amir Jahangiri[1] **and Vladislav Orekhov**[1,*]

[1]Department of Chemistry and Molecular Biology, Swedish NMR Centre, University of Gothenburg, Gothenburg, 40530, Sweden
[*]vladislav.orekhov@nmr.gu.se

Contents

1 Supplementary Methods **S2**
1.1 Python code for 2D WNN for Echo (or Anti-Echo) Reconstruction S2
1.2 Python code for 2D WNN for uncertainty estimation S3
2 Supplementary Results **S4**

List of Figures

1 2D ^{1}H-^{15}N — TROSY spectra of Malt, including the normal, the Echo and Anti-Echo reconstruction using MR-Ai and CS, and NUS reconstruction using CS S4
2a 2D ^{1}H-^{15}N — TROSY spectra of Tau, including the normal, the Echo and Anti-Echo reconstruction using MR-Ai and CS, and NUS (50%) reconstruction using CS S5
2b Zoomed-in view of the highly overlapping region around 8.25 ppm and 125 ppm in Figure 2a with the same contour level. S6
3 2D ^{1}H-^{15}N — HSQC spectra of Azurin, including the normal, the Echo and Anti-Echo reconstruction using MR-Ai and CS, and NUS reconstruction using CS S7
4 2D ^{1}H-^{15}N — HSQC spectra of Ubiquitin, including the normal, the Echo and Anti-Echo reconstruction using MR-Ai and CS, and NUS reconstruction using CS S8
5 Predicted uncertainty (pink) and actual error (red) for 2D ^{1}H-^{15}N — TROSY reconstructed Echo, Anti-Echo, and NUS spectra of Malt1 using MR-Ai and CS. All spectra are normalized to the reconstructed highest pick intensity.S9
6 Predicted uncertainty (pink) and actual error (red) for 2D ^{1}H-^{15}N — TROSY reconstructed Echo, Anti-Echo, and NUS spectra of Tau using MR-Ai and CS. All spectra are normalized to the reconstructed highest pick intensity.S9
7 Predicted uncertainty (pink) and actual error (red) for 2D ^{1}H-^{15}N — HSQC reconstructed Echo, Anti-Echo, and NUS spectra of Azurin using MR-Ai and CS. All spectra are normalized to the reconstructed highest pick intensity. S10
8 Predicted uncertainty (pink) and actual error (red) for 2D ^{1}H-^{15}N — HSQC reconstructed Echo, Anti-Echo, and NUS spectra of Ubiquitin using MR-Ai and CS. All spectra are normalized to the reconstructed highest pick intensity. S10
9 Traditional reference-based evaluation metrics. Root-mean-square deviation (*RMSD*) and the correlation coefficient (R_S^2) between reference and reconstruction spectra using different methods for Malt1 (a), Tau (b), Azurin (c), and Ubiquitin (d) proteins, and three reconstructions methods: MR-Ai for Echo (Anti-Echo) data; CS for Echo (Anti-Echo) data; CS for time equivalent 50% NUS. S11
10 Predicted reference-free spectrum quality metric, pSQ. Boxplots of the predicted uncertainties reconstruction spectra using different methods for Malt1 (a), Tau (b), Azurin (c), and Ubiquitin (d) proteins, and three reconstructions methods: MR-Ai for Echo (Anti-Echo) data; CS for Echo (Anti-Echo) data; CS for time equivalent 50% NUS. S11

1 Supplementary Methods

1.1 Python code for 2D WNN for Echo (or Anti-Echo) Reconstruction

```
import tensorflow as tf
import numpy as np

def WNN_2D(Size=32,filter=50,LR=0.0001):
    I = np.log2(Size).astype(int)

    input = tf.keras.layers.Input(shape = [2*Size-1, 4*Size-1])

    x = tf.keras.layers.Reshape((input.shape[1],input.shape[2],1))(input)
    x = tf.keras.layers.Conv2D(filter, kernel_size = (2,4))(x)
    x = tf.keras.activations.relu(x)

    for dil in range(1,I-1):
        x = tf.keras.layers.Conv2D(filter, kernel_size = (2,2),
                                   dilation_rate = (2**dil,2**(dil+1)))(x)
        x = tf.keras.activations.relu(x)

    x = tf.keras.layers.Conv2D(1, kernel_size=(2,2),
                               dilation_rate = (2**(dil+1),2**(dil+2)))(x)
    output = tf.keras.layers.Reshape((Size, 2*Size))(x)

    model = tf.keras.Model(inputs = input, outputs = output)
    model.compile(loss = tf.keras.losses.MeanSquaredError(),
                  optimizer = tf.keras.optimizers.Adam(learning_rate = LR))
    return model
```

1.2 Python code for 2D WNN for uncertainty estimation

```
import tensorflow as tf
import tensorflow_probability as tfp
import numpy as np

def NLL(y, distr):
    return -distr.log_prob(y)

def my_dist(params):
    return tfp.distributions.Normal(loc=params[...,0], scale=params[...,1])

def WNN_2D_Uncertainty(Size=32,filter=50,LR=0.001):
    I = np.log2(Size).astype(int)

    input = tf.keras.layers.Input(shape = [2*size-1,4*size-1])

    x = tf.keras.layers.Reshape((input.shape[1],input.shape[2],1))(input)
    x = tf.keras.layers.Conv2D(filter, kernel_size = (2,4))(x)
    x = tf.keras.activations.relu(x)

    for dil in range(1,I-1):
        x = tf.keras.layers.Conv2D(filter, kernel_size = (2,2),
                                   dilation_rate = (2**dil,2**(dil+1))(x)
        x = tf.keras.activations.relu(x)

    x = tf.keras.layers.Conv2D(filter, kernel_size=(2,2),
                               dilation_rate = (2**(dil+1),2**(dil+2)))(x)
    std = 1e-3 + tf.keras.activations.relu(1e-2 * x)
    output_std = tf.keras.layers.Reshape((size, 2*size))(std)

    input_2 = tf.keras.layers.Input(shape = [size, 2*size])
    mean = tf.keras.layers.Reshape((input_2.shape[1],input_2.shape[2],1))(input_2)
    output_mean = tf.keras.layers.Reshape((size, 2*size))(mean)

    params = tf.keras.layers.Concatenate()([mean,std])
    dist = tfp.layers.DistributionLambda(my_dist)(params)

    model = tf.keras.Model(inputs = [input,input_2], outputs = dist)
    model.compile(loss = NLL,
                  optimizer = tf.keras.optimizers.Adam(learning_rate = LR))
    model_std = tf.keras.Model(inputs=input, outputs=output_std)
    model_mean = tf.keras.Model(inputs=input_2, outputs=output_mean)
    return [model, model_std, model_mean]
```

2 Supplementary Results

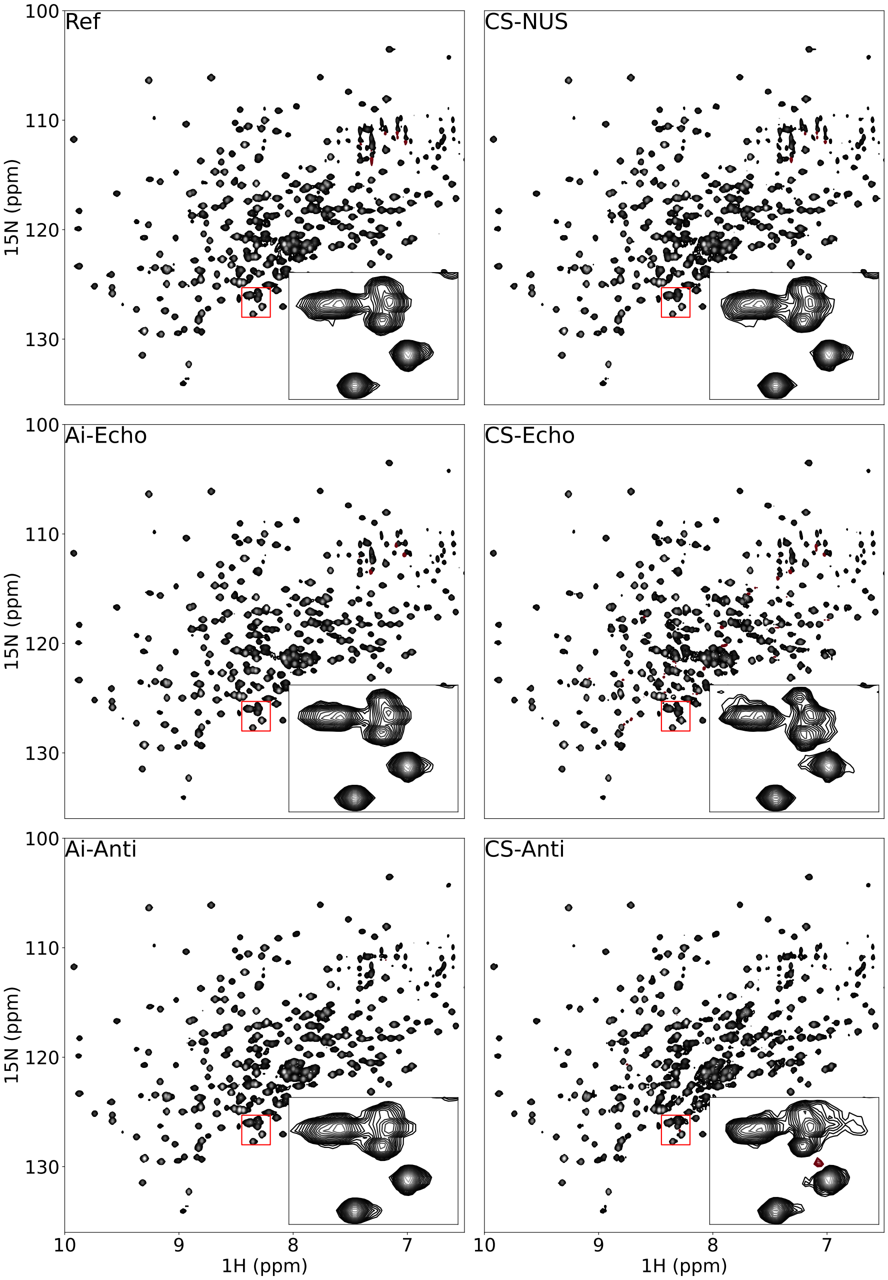

Supplementary Figure 1. 2D ^{1}H-^{15}N — TROSY spectra of Malt, including the normal, the Echo and Anti-Echo reconstruction using MR-Ai and CS, and NUS reconstruction using CS

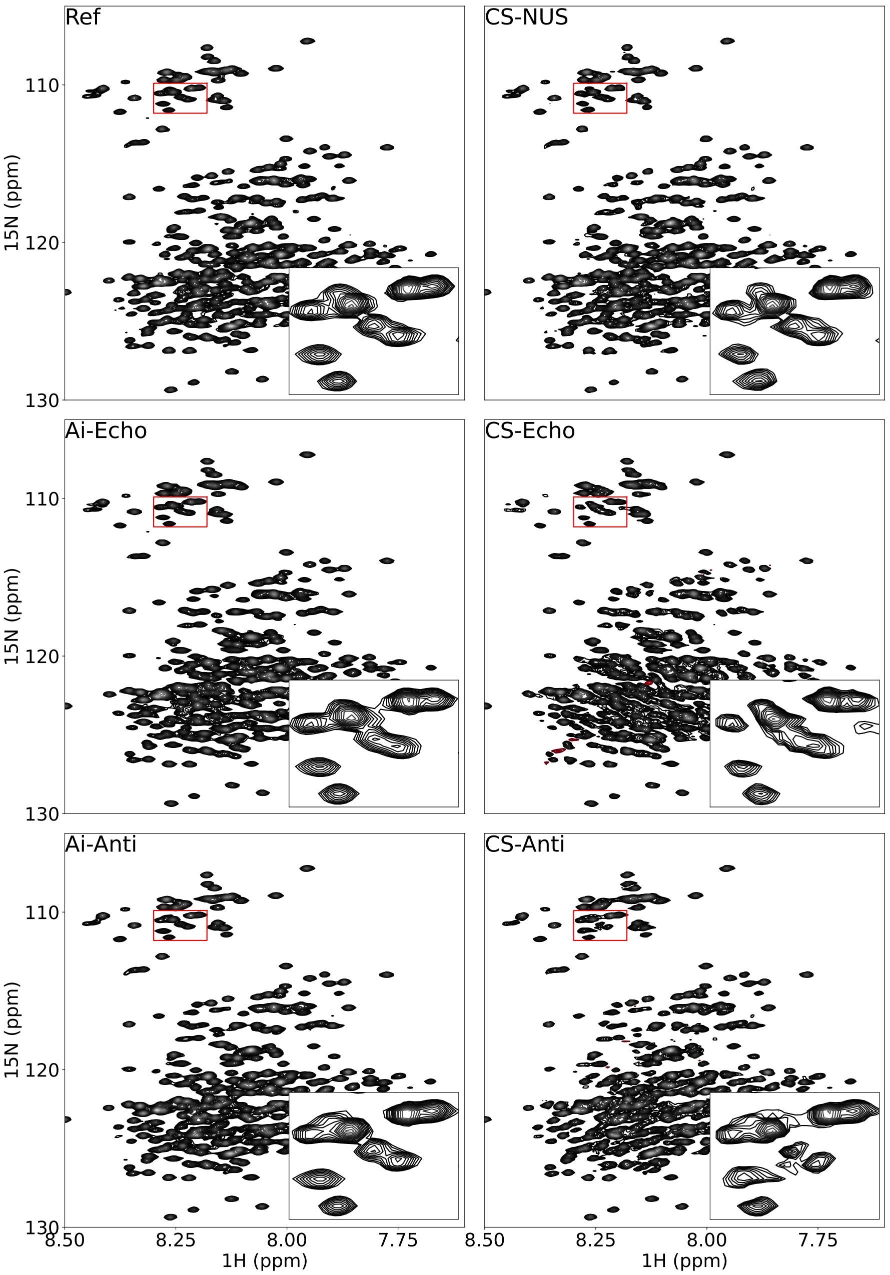

Supplementary Figure 2a. 2D ^{1}H-^{15}N — TROSY spectra of Tau, including the normal, the Echo and Anti-Echo reconstruction using MR-Ai and CS, and NUS (50%) reconstruction using CS

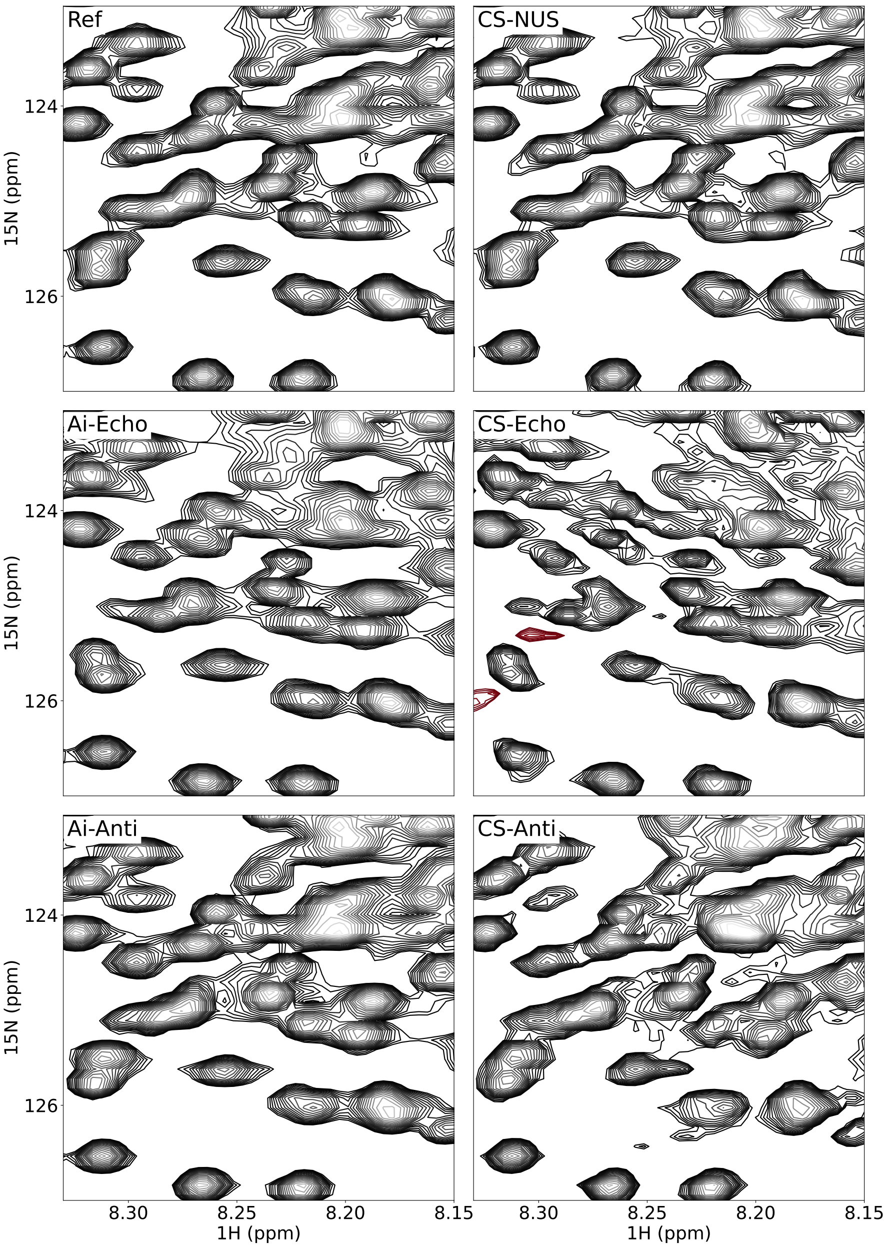

Supplementary Figure 2b. Zoomed-in view of the highly overlapping region around 8.25 ppm and 125 ppm in Figure 2a with the same contour level.

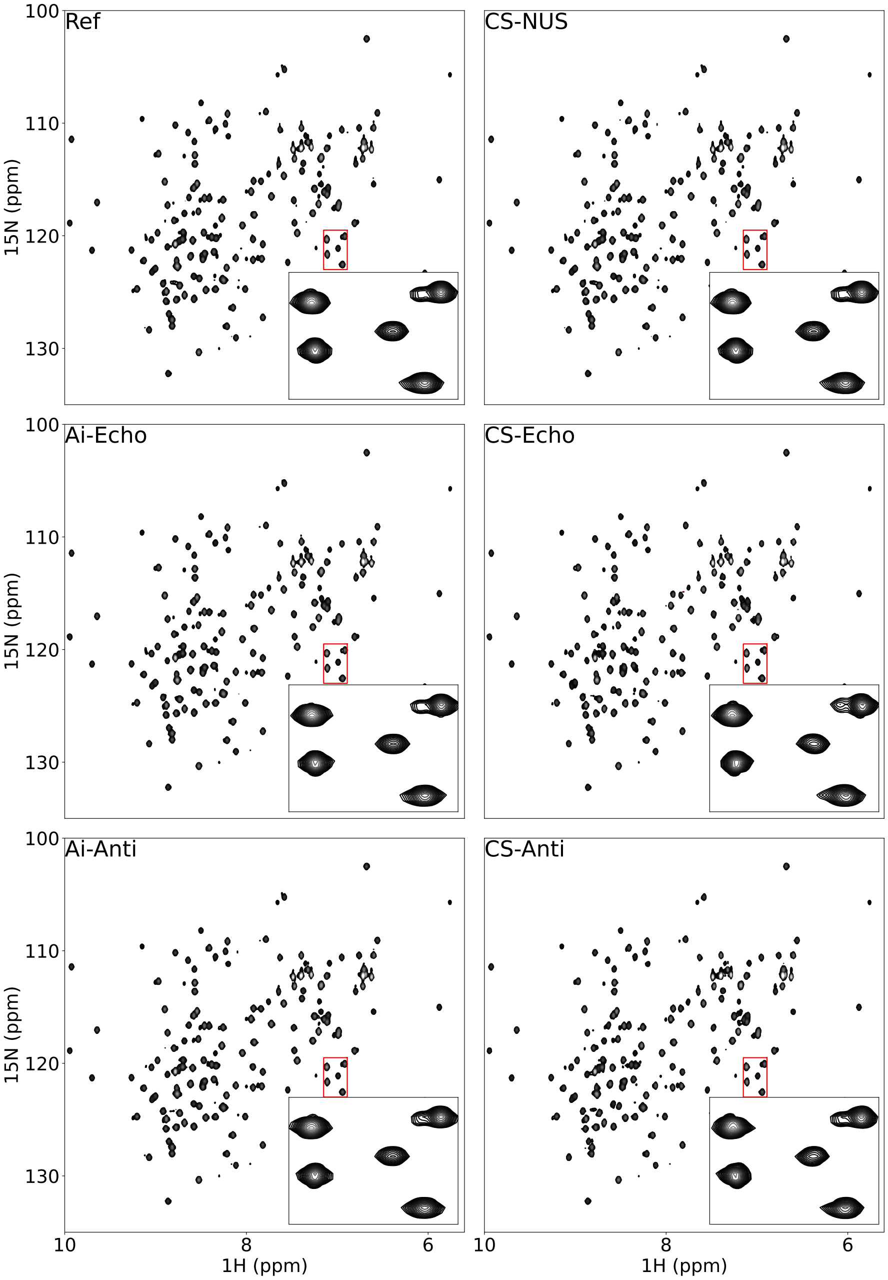

Supplementary Figure 3. 2D ^{1}H-^{15}N — HSQC spectra of Azurin, including the normal, the Echo and Anti-Echo reconstruction using MR-Ai and CS, and NUS reconstruction using CS

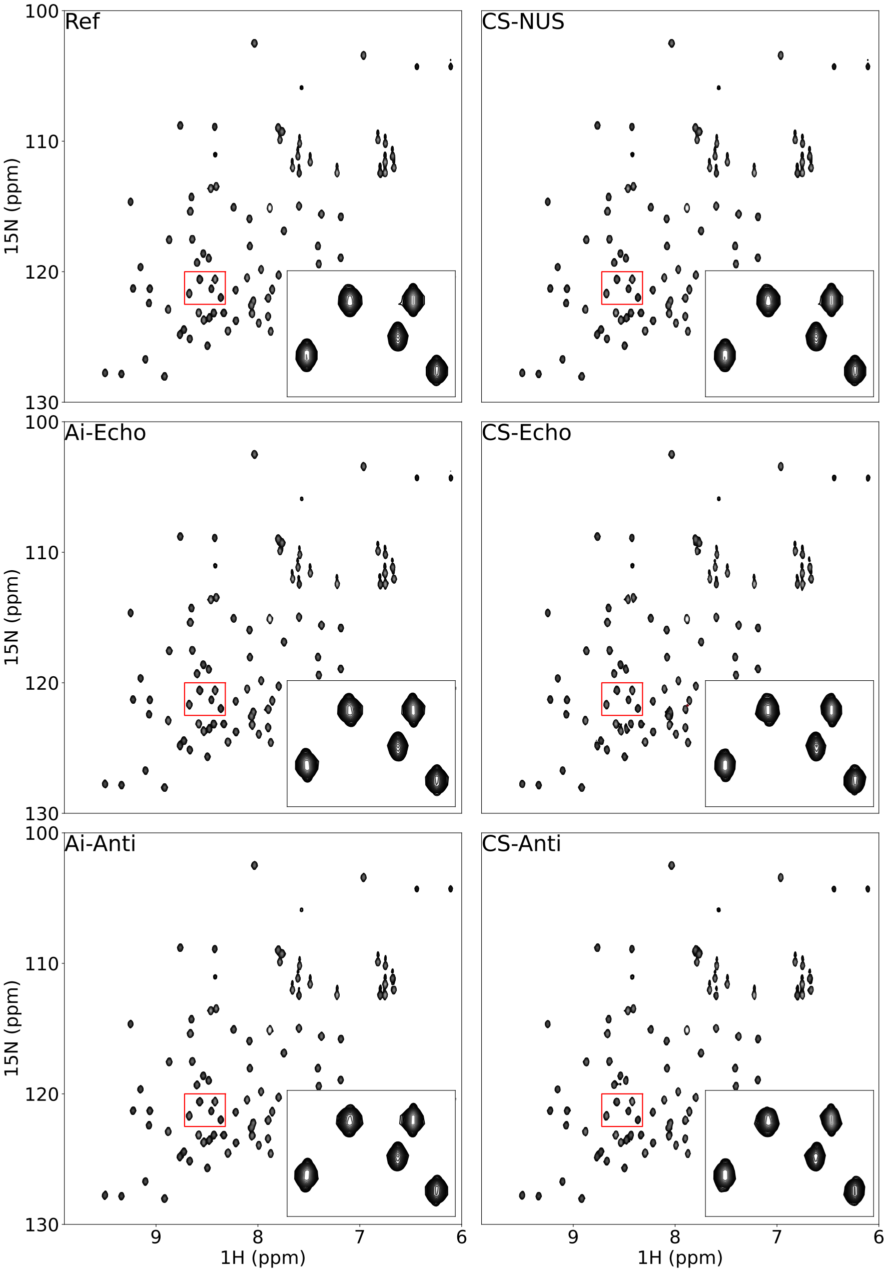

Supplementary Figure 4. 2D ^{1}H-^{15}N — HSQC spectra of Ubiquitin, including the normal, the Echo and Anti-Echo reconstruction using MR-Ai and CS, and NUS reconstruction using CS

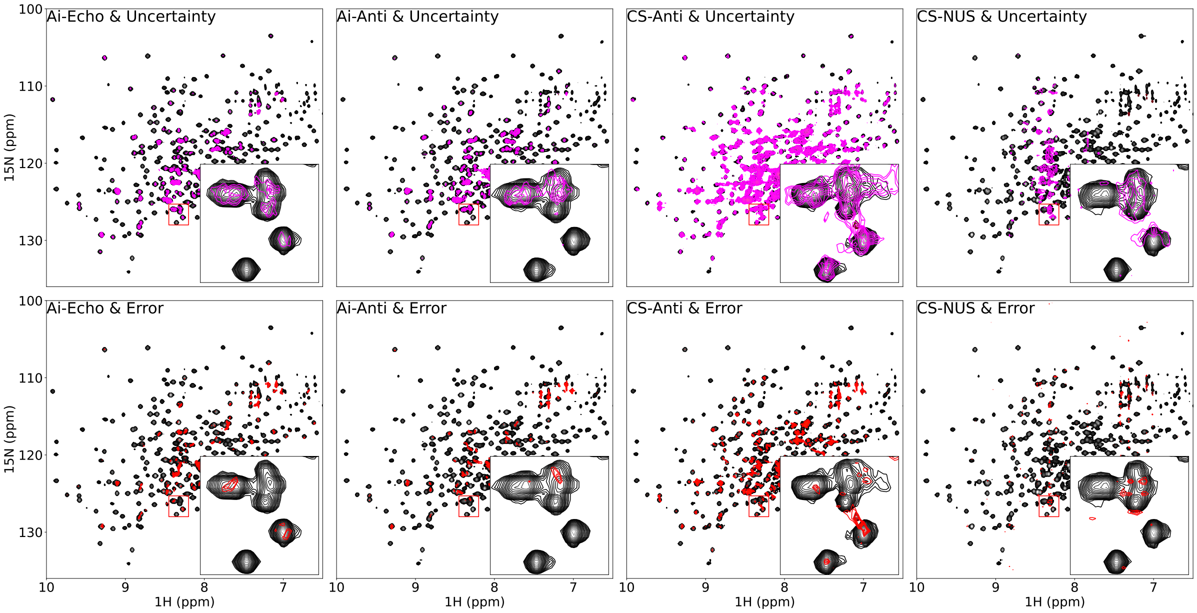

Supplementary Figure 5. Predicted uncertainty (pink) and actual error (red) for 2D ^{1}H-^{15}N — TROSY reconstructed Echo, Anti-Echo, and NUS spectra of Malt1 using MR-Ai and CS. All spectra are normalized to the reconstructed highest pick intensity.

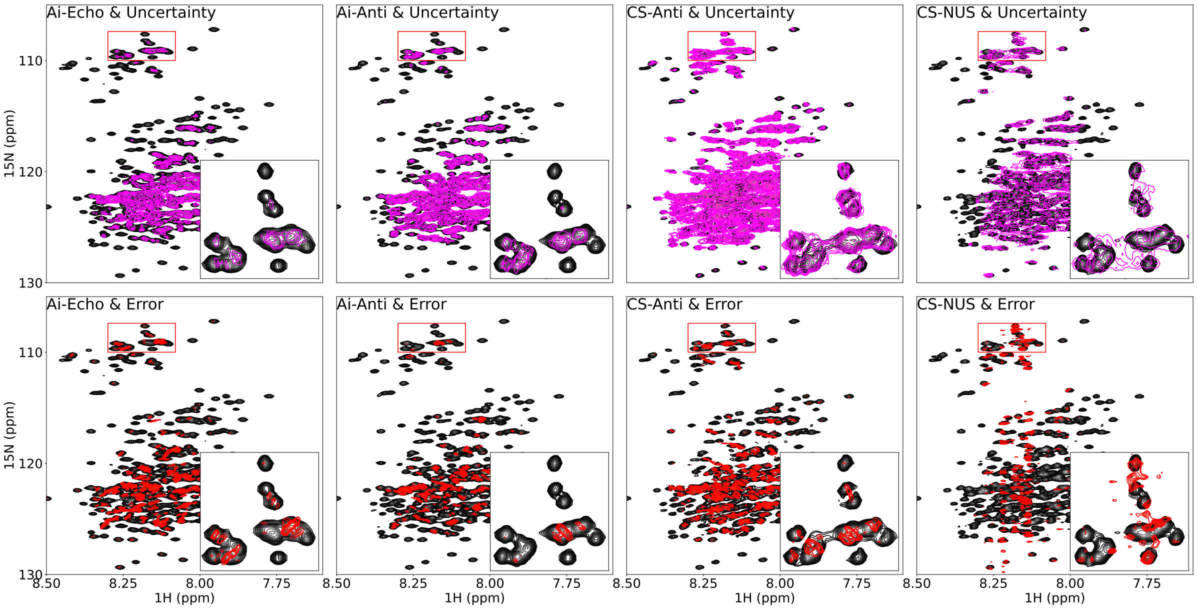

Supplementary Figure 6. Predicted uncertainty (pink) and actual error (red) for 2D ^{1}H-^{15}N — TROSY reconstructed Echo, Anti-Echo, and NUS spectra of Tau using MR-Ai and CS. All spectra are normalized to the reconstructed highest pick intensity.

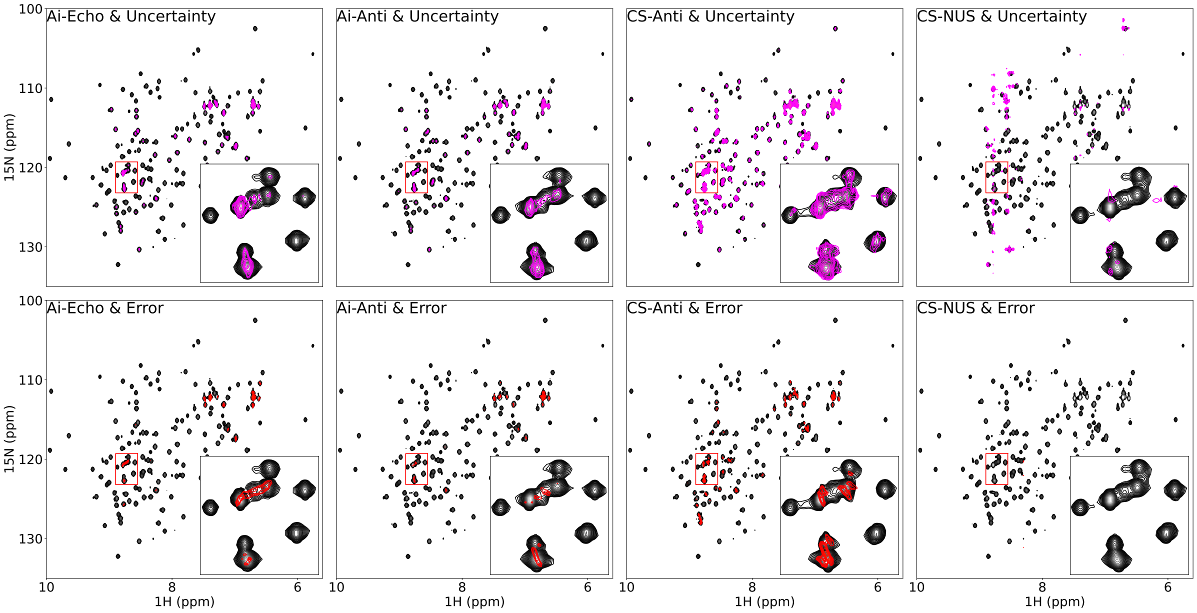

Supplementary Figure 7. Predicted uncertainty (pink) and actual error (red) for 2D ^{1}H-^{15}N — HSQC reconstructed Echo, Anti-Echo, and NUS spectra of Azurin using MR-Ai and CS. All spectra are normalized to the reconstructed highest pick intensity.

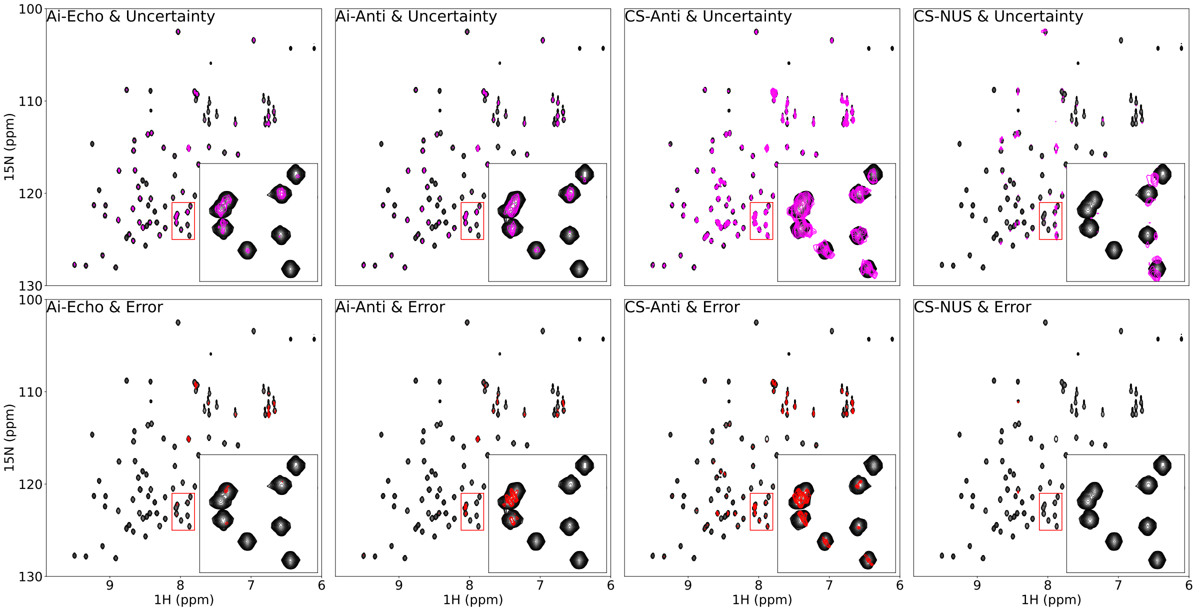

Supplementary Figure 8. Predicted uncertainty (pink) and actual error (red) for 2D ^{1}H-^{15}N — HSQC reconstructed Echo, Anti-Echo, and NUS spectra of Ubiquitin using MR-Ai and CS. All spectra are normalized to the reconstructed highest pick intensity.

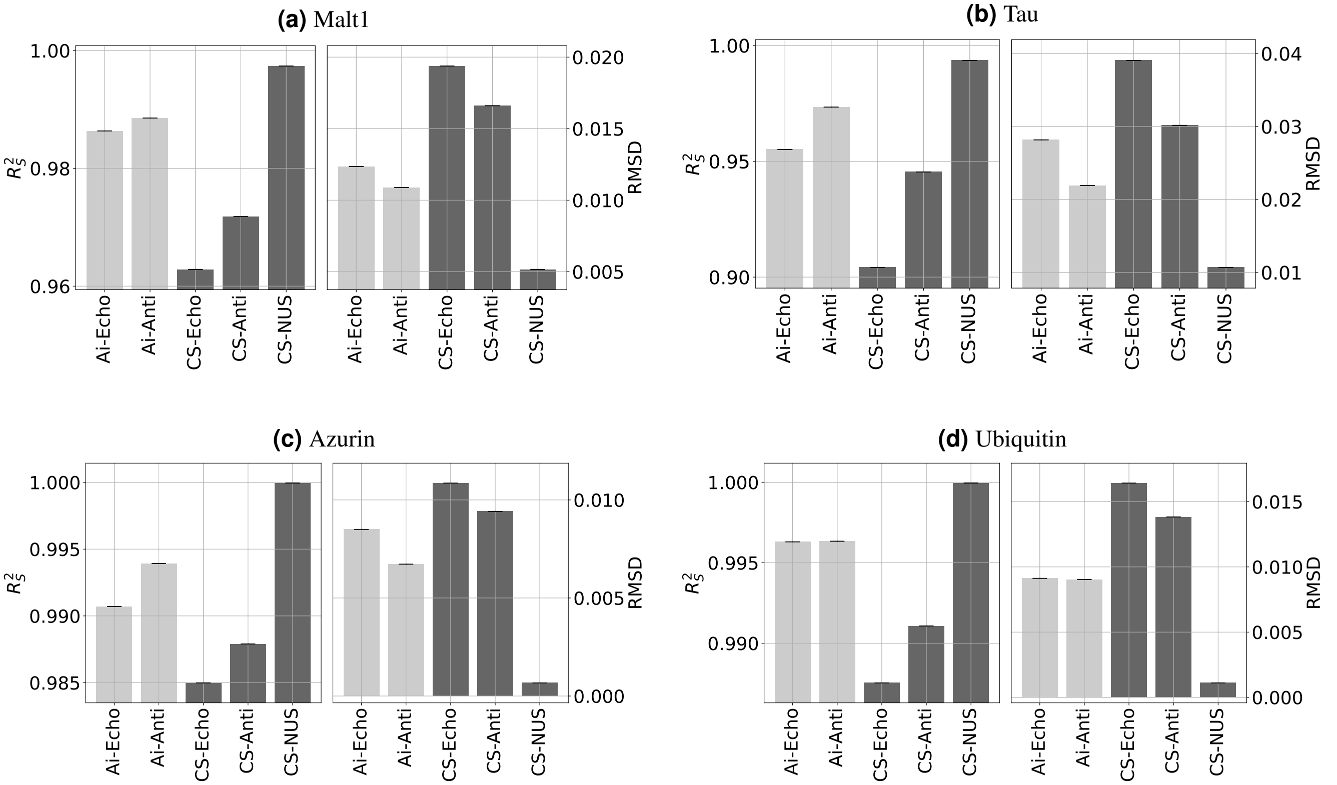

Supplementary Figure 9. Traditional reference-based evaluation metrics. Root-mean-square deviation (*RMSD*) and the correlation coefficient (R_S^2) between reference and reconstruction spectra using different methods for Malt1 (a), Tau (b), Azurin (c), and Ubiquitin (d) proteins, and three reconstructions methods: MR-Ai for Echo (Anti-Echo) data; CS for Echo (Anti-Echo) data; CS for time equivalent 50% NUS.

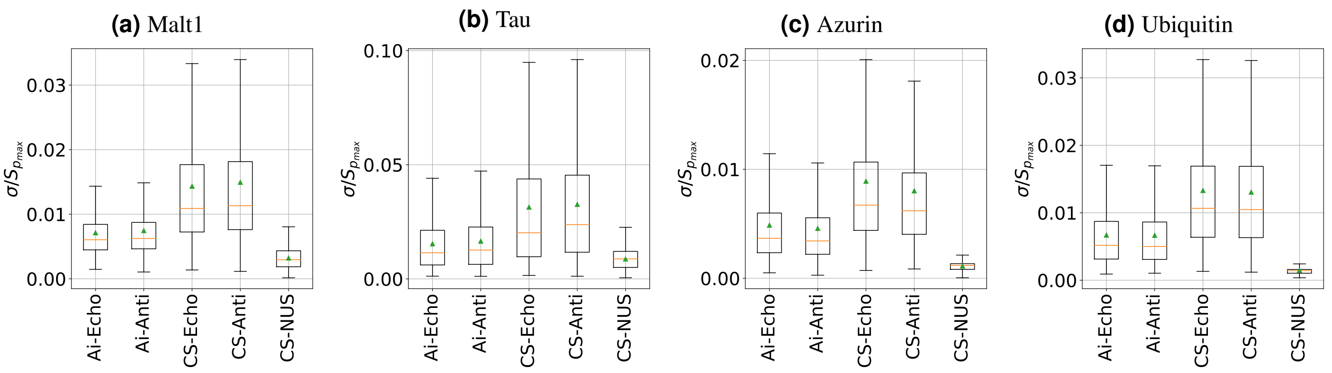

Supplementary Figure 10. Predicted reference-free spectrum quality metric, pSQ. Boxplots of the predicted uncertainties reconstruction spectra using different methods for Malt1 (a), Tau (b), Azurin (c), and Ubiquitin (d) proteins, and three reconstructions methods: MR-Ai for Echo (Anti-Echo) data; CS for Echo (Anti-Echo) data; CS for time equivalent 50% NUS.
